# Rab1A regulates anterograde melanosome transport by recruiting kinesin-1 to melanosomes through interaction with SKIP

**DOI:** 10.1038/srep08238

**Published:** 2015-02-04

**Authors:** Morié Ishida, Norihiko Ohbayashi, Mitsunori Fukuda

**Affiliations:** 1 Laboratory of Membrane Trafficking Mechanisms, Department of Developmental Biology and Neurosciences, Graduate School of Life Sciences, Tohoku University, Aobayama, Aoba-ku, Sendai, Miyagi 980-8578, Japan

## Abstract

Melanosomes are lysosome-related organelles in melanocytes that are transported from the perinucleus to the cell periphery by coordination between bidirectional (anterograde and retrograde) microtubule-dependent transport and unidirectional actin-dependent transport. Although the molecular machineries that mediate retrograde transport and actin-dependent transport have already been identified, little is known about the anterograde transport complex on microtubules in mammalian cells. Here we discovered that small GTPase Rab1A on melanosomes recruits SKIP/PLEKHM2 as a Rab1A-specific effector and that Rab1A, SKIP, and a kinesin-1/(Kif5b+KLC2) motor form a transport complex that mediates anterograde melanosome transport in melanocytes. Interestingly, Arl8, Arf-like small GTPase that also interacts with SKIP, is specifically localized at lysosomes and regulates their anterograde transport in melanocytes. Our findings suggest that the anterograde microtubule-dependent transport of melanosomes and lysosomes are differently regulated by independent cargo receptors, i.e., Rab1A and Arl8, respectively, but that a SKIP–kinesin-1 mechanism is responsible for the transport of both.

Melanosomes are cell-type-specific organelles that synthesize and preserve melanin pigments and are exclusively produced in pigment cells called melanocytes. Melanosomes are also known as members of a group of lysosome-related organelles (LROs), which share common characteristics with lysosomes and have diverse physiological functions that maintain homeostasis in metazoan cells^1^. Pigmentation of mammalian skin and hair requires not only the formation of melanosomes but their transport from the perinucleus to the cell periphery of melanocytes along two components of the cytoskeleton, namely microtubules and actin filaments^1^,^2^,^3^. Actually, defects in melanosome movements on actin filaments have been shown to cause human Griscelli syndrome (GS), which is characterized by hypopigmentation of hair and skin^4^. During the past few decades, a variety of key factors involved in melanosome transport have been identified by genetic analyses of patients with albinism and of coat color mutant mice^2^,^4^,^5^,^6^. Functional analyses of these factors have revealed the molecular mechanisms of actin-dependent and microtubule-dependent retrograde melanosome transport. A tripartite protein complex composed of small GTPase Rab27A (GS2 and *ashen* gene product), its specific effector Slac2-a/melanophilin (GS3 and *leaden* gene product), and an actin-based motor myosin Va (GS1 and *dilute* gene product) regulates actin-dependent melanosome transport^4^,^7^,^8^,^9^,^10^, while both melanoregulin (*dilute suppressor* gene product) and Rab36 regulate retrograde melanosome transport through interaction with RILP and p150^Glued^, a subunit of the dynein–dynactin motor complex^11^,^12^. Interestingly, however, no mammalian pigmentary disorders, in which impaired regulation of microtubule-dependent anterograde melanosome transport (hereafter simply referred to as anterograde melanosome transport) has been implicated, have ever been reported, and as a result the molecular mechanism responsible for it largely remains to be elucidated.

We recently showed by performing a comprehensive analysis of the Rab family small GTPases that Rab1A, originally described as a Golgi-resident Rab, is localized at mature melanosomes and involved in anterograde melanosome transport in melanocytes^13^. In general, microtubule-dependent anterograde organelle transport is regulated by a superfamily of molecular motors, the kinesin superfamily proteins (Kifs)^14^,^15^, and direct or indirect interactions between Rab proteins and Kifs^16^ have been reported in other types of organelle transport. However, the functional link between Rab1A and Kifs in melanosome transport has never been investigated.

Here we report finding that Rab1A indirectly recruits the kinesin-1 motor (composed of two Kif5b heavy chains and two light chains) to mature melanosomes through interaction with a Rab1A effector molecule, SKIP (SifA and kinesin-interacting protein, also known as PLEKHM2), which was previously characterized as a kinesin-1 and Arl8 binding protein involved in lysosome transport toward the cell periphery^17^,^18^. Based on our findings below we discuss the possible mechanisms by which a SKIP–kinesin-1 complex differentially transports two related types of organelles, melanosomes, which are LROs, and lysosomes, in melanocytes.

## Results

### Comprehensive analysis of the interaction between Rab1A and the tail domains of kinesin motor proteins by yeast two-hybrid assays

Most Kifs consist of a microtubule-binding motor domain and a tail domain, which is required to recognize a cargo receptor(s)^14^,^15^. We initially hypothesized that Rab1A directly interacts with the tail domain of certain Kifs for two reasons, first, because the tail domain of some Kifs has been shown to directly interact with certain Rabs, e.g., Kif20A/Rabkinesin-6 with Rab6A/B^19^, Kif16B with Rab14^20^ and Kif13A with Rab11^21^ and, second, because the tail domain of most Kifs contains coiled-coil domains, which often serve as Rab-binding sites^22^. To test our hypothesis, we first cloned cDNAs of the tail domains of 43 members of the Kif family found in the mouse genome (i.e., the tail domains of Kif1–27 and Kifc1–3) and subcloned them into the pAct2 vector for yeast two-hybrid screening^23^,^24^. We then performed yeast two-hybrid assays to test all the possible combinations of interactions between a constitutively active (CA) Rab1A mutant, which mimics the GTP-bound form, or a constitutively negative (CN) Rab1A mutant, which mimics the GDP-bound form, and the tail domain of each Kif (Supplementary Fig. S1). However, no positive interactions between Rab1A and the tail domain of any of the Kifs was detected (Supplementary Fig. S1), whereas a positive control, the Rusc2 RUN domain specifically interacted with Rab1A(CA) under the same experimental conditions^25^. We therefore concluded that Kif proteins themselves are unlikely to function as Rab1A effectors during anterograde melanosome transport in melanocytes.

### Identification of SKIP as a specific Rab1A effector and as a novel regulator of melanosome transport in melanocytes

**Figure 1:**
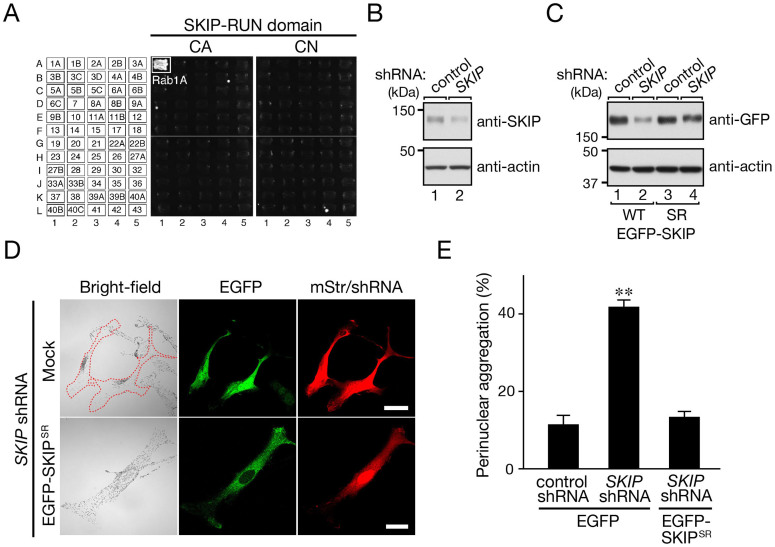
Functional involvement of SKIP in melanosome distribution in melanocytes. (A) Rab binding specificity of the RUN domain of SKIP as revealed by yeast two-hybrid panels^22^,^54^. Yeast cells containing pGBD-C1 plasmid expressing each of the 60 constitutively active/constitutively negative (CA/CN) Rabs (positions indicated in the left panels) and pAct2-SKIP-RUN were streaked on SC-AHLW and incubated at 30°C for 1 week. Note the specific, GTP-dependent interaction between SKIP-RUN and Rab1A. (B) Knockdown of endogenous SKIP protein in melan-a cells with the specific *SKIP* shRNA. The positions of the molecular mass markers are shown on the left. (C) Characterization of an shRNA-resistant (SR) SKIP^SR^ mutant. (D) Knockdown of SKIP in melanocytes resulted in perinuclear melanosome aggregation. Melan-a cells were cotransfected with pSilencer-mStr-SKIP and either pEGFP-C1 (top row) or pEGFP-C1-SKIP^SR^ (bottom row). Cells exhibiting perinuclear melanosome aggregation are outlined with broken lines. Note the clear perinuclear melanosome aggregation in the SKIP-deficient cells (top left panel), whereas re-expression of SKIP^SR^ completely restored peripheral melanosome distribution (bottom left panel). (E) Quantification of the results shown in (D). The results are expressed as the percentages of transfected cells that exhibited perinuclear melanosome aggregation, and the bars represent the means and S.E. of the data from three independent experiments (n >50). **, *p* <0.01 in comparison with the control cells (Dunnett's test). Scale bars, 20 μm.

After rejecting our first hypothesis because no interaction between Rab1A and Kifs had been detected (Supplementary Fig. S1), we hypothesized that an indirect interaction between Rab1A and Kifs occurs via a linker protein that binds both Rab1A and Kif. Actually, several Rab effectors have been shown to bind Kifs and to be involved in organelle transport^26^,^27^,^28^,^29^,^30^. To test our second hypothesis, we screened for a Rab1A-specific effector molecule by yeast two-hybrid assays using putative Rab-binding domains, e.g., a coiled-coil domain and a RUN domain, in the mouse or human genome as bait^22^,^25^ (and M. F., unpublished data). During the course of this screening, we succeeded in identifying SKIP (SifA and kinesin-interacting protein)^17^ as a specific Rab1A-binding protein by using the RUN domain of SKIP as bait (Fig. 1A). SKIP is also known as PLEKHM2 (pleckstrin homology domain containing, family M (with RUN domain) member 2), which is characterized by an N-terminal RUN domain and C-terminal PH domain. As shown in Fig. 1A, the N-terminal RUN domain-containing region of SKIP (1–282 amino acids) specifically recognized the CA form of Rab1A, but it did not interact with its CN form or with the CA/CN forms of any of the other 59 Rabs tested. It should be noted that SKIP has recently been shown to simultaneously interact with Arf-like GTPase Arl8 on lysosomes and kinesin-1 and that the resulting Arl8–SKIP–kinesin-1 motor complex transports lysosomes to the cell periphery in HeLa cells^18^. Moreover, because melanosomes are LROs^1^, it seemed highly possible that SKIP also functions as a regulator of anterograde melanosome transport.

Because both Arl8 and Rab1A interact with the N-terminal RUN domain-containing region of SKIP, we first mapped their binding sites by using a series of deletion mutants (Supplementary Fig. S2A). The results of the yeast two-hybrid assays showed that the two GTPases interacted with similar N-terminal RUN domain-containing regions of SKIP, suggesting that their binding to SKIP is mutually exclusive and that they function as an independent cargo receptor (see below for details). Consistent with previous reports^18^,^31^, we observed that monomeric Strawberry (mStr)-tagged Arl8b was clearly localized at lysosomes even in melanocytes (mouse melan-a cells), but none was present on mature melanosomes (Supplementary Fig. S3A, arrows). In addition, when we expressed SKIP together with Arl8b in melanocytes, the two proteins were colocalized at Lamp1-positive lysosomes, and peripheral lysosome enrichment was often observed (Supplementary Fig. S3B, arrowheads). By contrast, no change in melanosome distribution was observed in SKIP/Arl8b-expressing cells (Supplementary Fig. S3B, far right panel, and C). These results taken together indicated that the primary role of Arl8b in melanocytes is regulation of anterograde lysosome transport, the same as in HeLa cells, and thus that Arl8 is highly unlikely to be a cargo receptor for anterograde melanosome transport in melanocytes.

To determine whether the Rab1A-binding protein SKIP is also involved in anterograde melanosome transport, we knocked down endogenous SKIP protein with a specific shRNA (Fig. 1B). As shown in Fig. 1D (top row), SKIP knockdown resulted in significant perinuclear melanosome aggregation, the same as Rab1A knockdown did^13^. Moreover, re-expression of an shRNA-resistant form of SKIP (SKIP^SR^) in SKIP-knockdown cells (Fig. 1C) completely restored the peripheral melanosome distribution (Fig. 1D, bottom row, and E), thereby excluding the possibility of an off-target effect of the *SKIP* shRNA. These results indicated that SKIP is involved in anterograde melanosome transport in melanocytes as well as in lysosome transport.

### Knockdown of kinesin-1 resulted in perinuclear melanosome aggregation

**Figure 2:**
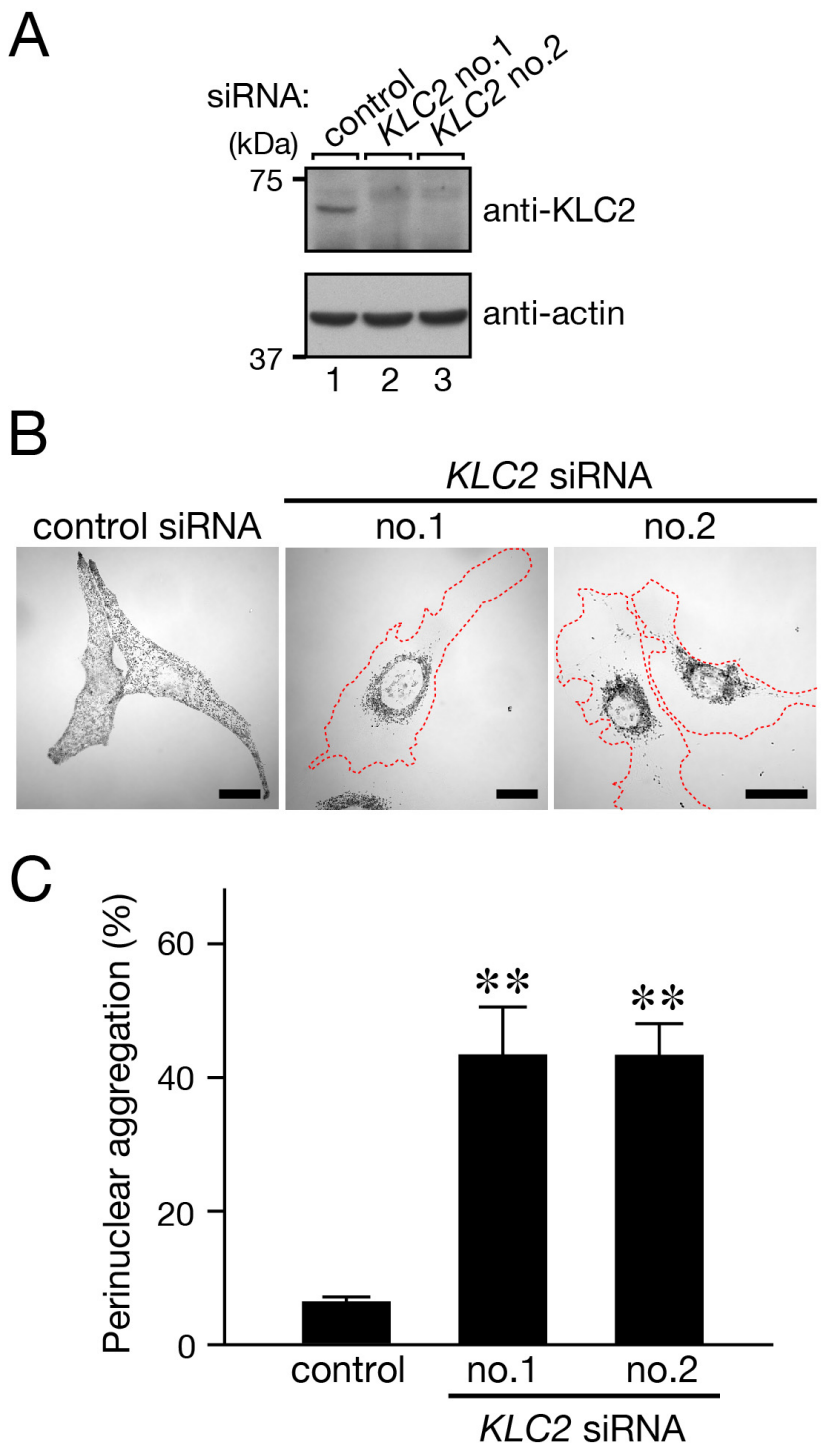
Effect of KLC2 knockdown in melanocytes on melanosome distribution. (A) Knockdown of endogenous KLC2 protein in melan-a cells with specific *KLC2* siRNAs. The positions of the molecular mass markers are shown on the left. (B) Knockdown of KLC2 in melanocytes resulted in perinuclear melanosome aggregation. Melan-a cells were treated with a control siRNA (left panel) or *KLC2* siRNAs (right two panels). Cells exhibiting perinuclear melanosome aggregation are outlined with broken lines. Note the clear perinuclear melanosome aggregation in the KLC2-deficient cells (right two panels). Scale bars, 20 μm. (C) Quantification of the results shown in (B). The results are expressed as the percentages of cells that exhibited perinuclear melanosome aggregation, and the bars represent the means and S.E. of the data from three independent experiments (n >100). **, *p* <0.01 in comparison with the control cells (Dunnett's test).

If SKIP recruits kinesin-1 in anterograde melanosome transport the same as in lysosome transport^18^, functional ablation of kinesin-1 should phenocopy Rab1A-deficient or SKIP-deficient melanocytes. In the next set of experiments, we attempted to knock down each subunit of kinesin-1 in melanocytes with specific siRNAs. Kinesin-1 is a typical member of the kinesin family and composed of two heavy chains (Kif5) and two light chains (KLC)^14^,^15^. The N-terminal WD motifs of SKIP have been shown to directly interact with kinesin light chain 2 (KLC2) to form a complex with kinesin-1^18^,^32^,^33^. Because KLC2 was endogenously expressed in melanocytes, we first investigated the effect of RNAi-mediated KLC2 knockdown on melanosome distribution (Fig. 2A). As expected, knockdown of KLC2 by two independent siRNAs resulted in significant perinuclear melanosome aggregation (Fig. 2B, C), the same as Rab1A knockdown^13^ and SKIP knockdown did (Fig. 1D, E).

**Figure 3:**
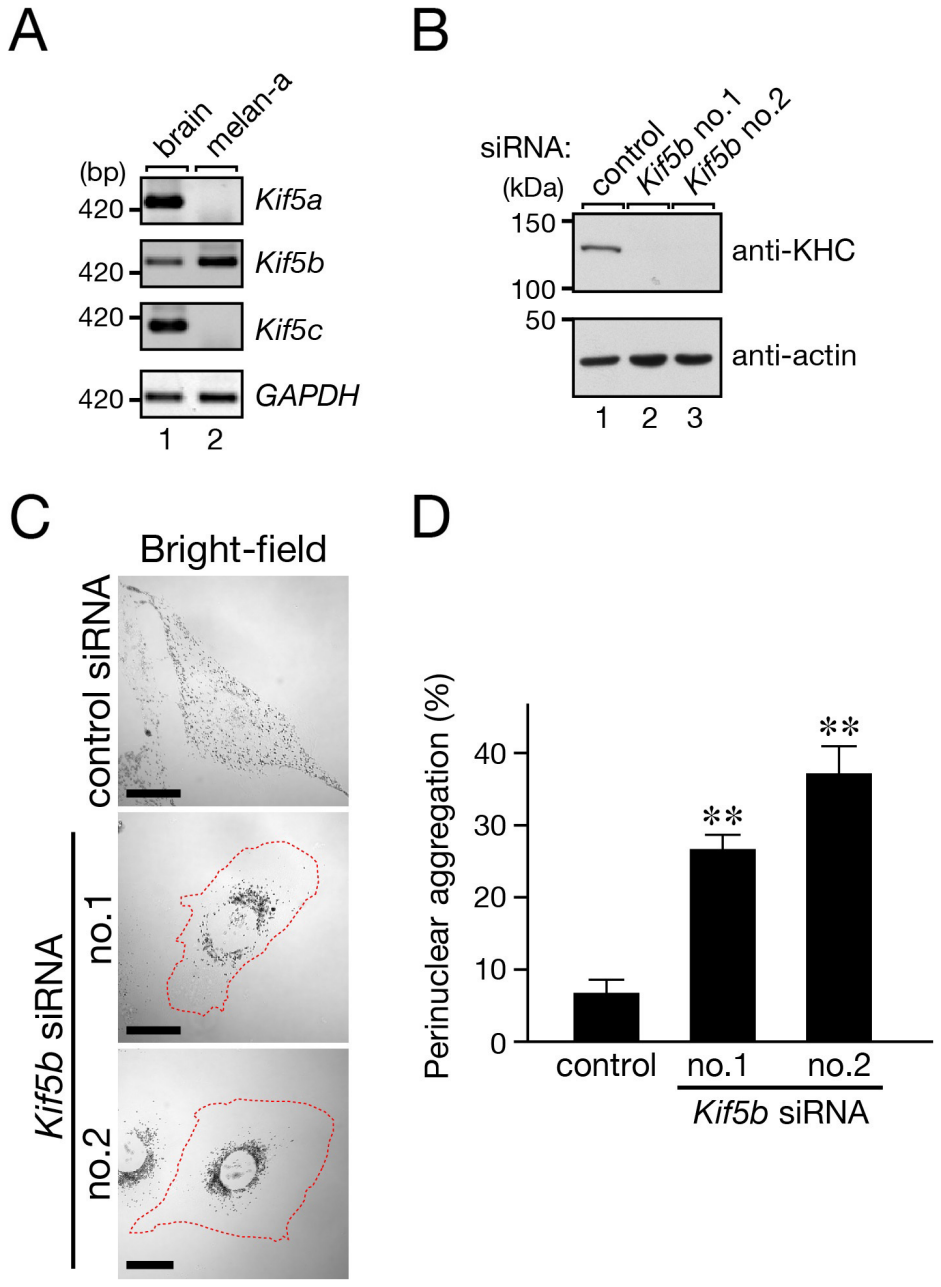
Effect of Kif5b knockdown in melanocytes on melanosome distribution. (A) Expression of *Kif5b* mRNA and not of *Kif5a* or *Kif5c* mRNA in melan-a cells as revealed by an RT-PCR analysis. The results are shown in the form of white/black-reversal images. *GAPDH* mRNA expression (bottom panel) is shown as a loading control to ensure that equivalent amounts of first-strand cDNA were used for the RT-PCR analysis. The size of the molecular mass markers (bp, base pairs) is shown on the left side of the panel. (B) Knockdown of endogenous Kif5b protein in melan-a cells with the specific *Kif5b* siRNAs. The positions of the molecular mass markers are shown on the left. (C) Knockdown of Kif5b in melanocytes resulted in perinuclear melanosome aggregation. Melan-a cells were treated with a control siRNA (top panel) or *Kif5b* siRNAs (bottom two panels). Cells exhibiting perinuclear melanosome aggregation are outlined with a broken line. Note the clear perinuclear melanosome aggregation in the Kif5b-deficient cells (bottom two panels). Scale bars, 20 μm. (D) Quantification of the results shown in (C). The results are expressed as the percentages of cells that exhibited perinuclear melanosome aggregation, and the bars represent the means and S.E. of the data from four independent experiments (n >100). **, *p* <0.01 in comparison with the control cells (Dunnett's test).

Next, we turned our attention to the kinesin heavy chains (Kif5) that function in anterograde melanosome transport. There are three different isoforms of Kif5, named Kif5a–c, in mice, and the results of the RT-PCR analysis indicated that only Kif5b, and not Kif5a or Kif5c, was expressed in melan-a cells (Fig. 3A). When we specifically knocked down Kif5b isoform in these cells with two independent siRNAs (Fig. 3B), perinuclear melanosome aggregation occurred (Fig. 3C, D), a finding that was consistent with a recent report on human melanoma cells^34^. These results allowed us to conclude that the kinesin-1 complex composed of Kif5b and KLC2 is actually involved in anterograde melanosome transport.

### Rab1A forms a functional transport complex with SKIP and kinesin-1 in melanocytes

**Figure 4:**
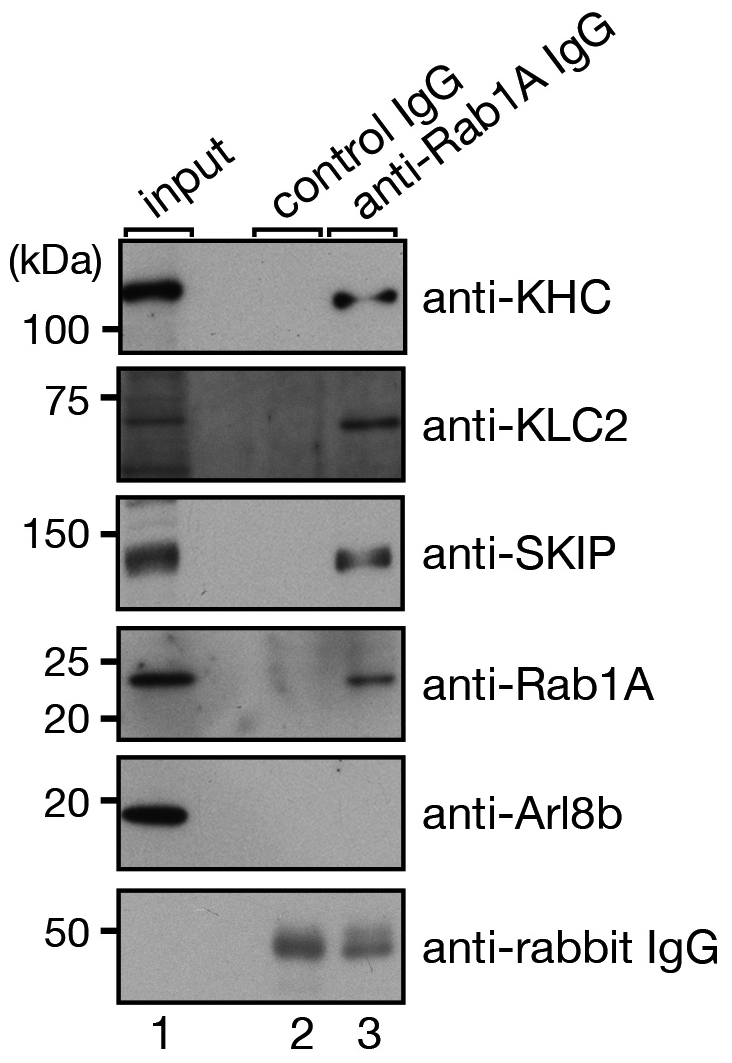
Formation of the Rab1A–SKIP–kinesin-1 complex in melan-a cells. Endogenous Rab1A molecules were immunoprecipitated from lysates of melan-a cells with anti-Rab1A specific IgG (lane 3) or control IgG (lane 2). Rab1A, SKIP, KLC2, kinesin heavy chain (KHC), and Arl8b were analyzed by immunoblotting with specific antibodies. The amount of IgG heavy chain used for immunoprecipitation was analyzed with an anti-rabbit IgG antibody (bottom panel). It should be noted that SKIP, KLC2, and KHC (Kif5b) were co-purified with Rab1A, but Arl8b was not. Input shows a 1/140 volume of the reaction mixture used for immunoprecipitation. The positions of the molecular mass markers are shown on the left.

**Figure 5:**
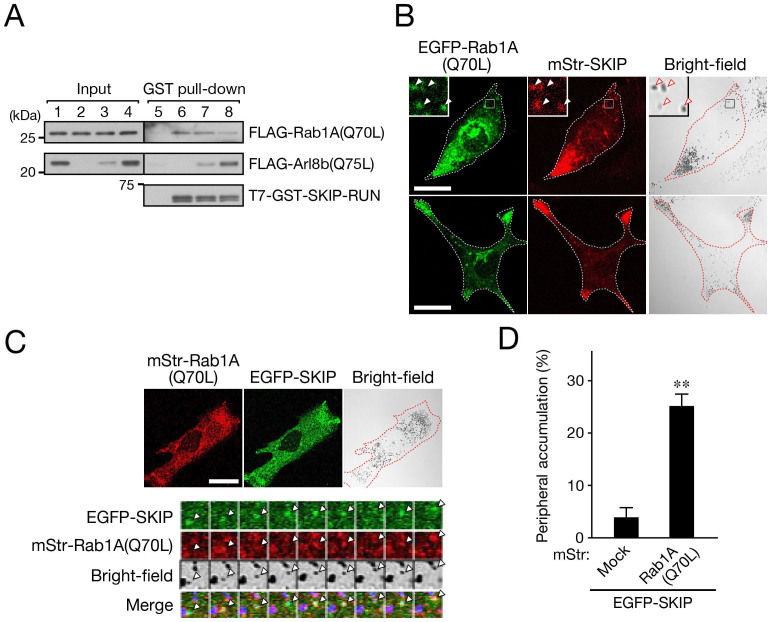
Rab1A on melanosomes interacts with SKIP and regulates anterograde melanosome transport. (A) Competition experiments revealed that Arl8b-binding and Rab1A-binding to SKIP are mutually exclusive. Glutathione Sepharose beads coupled with T7-GST-SKIP-RUN or beads alone were incubated with solutions containing FLAG-Rab1A(Q70L) and/or an increasing amount of FLAG-Arl8b(Q75L), and the proteins bound to the beads were analyzed by immunoblotting with the antibodies indicated. Input shows a 1/140 volume of the reaction mixture used for the GST pull-down assays. The positions of the molecular mass markers are shown on the left. Note that Rab1A binding to SKIP was reduced by an increasing amount of Arl8b (the signal intensity values of bound FLAG-Rab1A(Q70L) normalized to T7-GST-SKIP-RUN signals are 1.0 (lane 6), 0.95 (lane 7), and 0.36 (lane 8)). (B) Typical images of melan-a cells expressing EGFP-Rab1A(Q70L) together with mStr-SKIP are shown. The insets are magnified views of the boxed areas showing that Rab1A colocalized with SKIP at melanosomes (arrowheads). Note that cells expressing EGFP-Rab1A(Q70L) together with mStr-SKIP exhibited peripheral melanosome accumulation. (C) Live cell images of melan-a cells expressing EGFP-SKIP and mStr-Rab1A(Q70L), and montage images showing colocalization of both EGFP-SKIP and mStr-Rab1A(Q70L) at a melanosome and moving with it toward the cell periphery (arrowheads). Images of the cells in (B) and (C) exhibiting peripheral melanosome accumulation are outlined with a broken line. Scale bars, 20 μm. (D) Quantification of the results shown in (C). The results are expressed as the percentages of transfected cells that exhibited peripheral melanosome accumulation, and the bars represent the means and S.E. of the data from three independent experiments (n >50). **, *p* <0.01 in comparison with the mStr- and EGFP-SKIP-expressing cells (Student's unpaired *t* test).

In the final set of experiments, we attempted to determine whether Rab1A actually regulates anterograde melanosome transport in melanocytes through formation of a Rab1A–SKIP–kinesin-1 complex. To do so, we first performed a coimmunoprecipitation assay with anti-Rab1A specific antibody in melan-a cells. Consistent with the results of the *in vitro* binding experiments (Fig. 1A)^18^,^32^,^33^, endogenous Rab1A was co-purified with SKIP, KLC2, and Kif5b molecules from lysates of melan-a cells (top three panels in Fig. 4). Intriguingly, however, Arl8b, another SKIP-binding protein^18^, was not co-purified with Rab1A at all (fifth panel in Fig. 4), indicating that melanocytes contain two different SKIP–kinesin-1 complexes, i.e., the Rab1A–SKIP–kinesin-1 complex on melanosomes and the Arl8–SKIP–kinesin-1 complex on lysosomes. This observation was highly consistent with the results of the binding experiments shown in Supplementary Fig. S2: the Rab1A-binding site and Arl8b-binding site on SKIP almost completely overlapped. In addition, the results of the competition experiments between Rab1A and Arl8b indicated that Rab1A binding to SKIP-RUN (amino acid residues 1–282) was clearly inhibited by the presence of an increasing amount of Arl8b protein (Fig. 5A), i.e., binding of Arl8b and Rab1A to SKIP is mutually exclusive. Therefore, Arl8b and Rab1A are likely to function as distinct, independent cargo receptors for SKIP–kinesin-1.

Next, we coexpressed the constitutively active Rab1A(Q70L) mutant with SKIP in melanocytes and observed their effect on melanosome distribution. Intriguingly, both Rab1A(Q70L) and SKIP were often colocalized with mature melanosomes (Fig. 5B, arrowheads in the insets), and the melanosomes clearly accumulated at the cell periphery in approximately 25% of the transfected cells (Fig. 5B, D). As far as we know, this is a previously unreported, novel phenotype, and we named it “peripheral melanosome accumulation”. In addition, time-lapse imaging clearly showed that both the mStr-Rab1A(Q70L) signals and EGFP-SKIP signals moved together with the melanosomes to the cell periphery (Fig. 5C).

**Figure 6:**
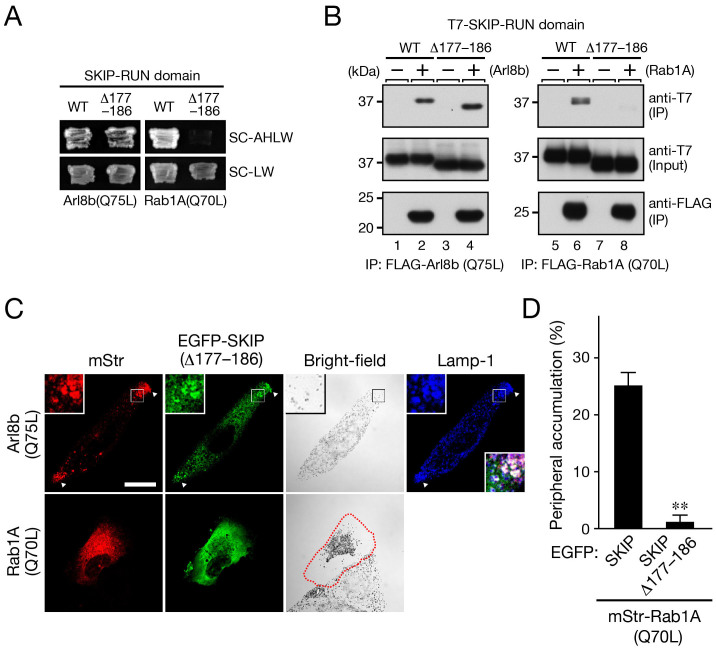
The Rab1A–SKIP interaction is necessary for anterograde melanosome transport. (A) Interaction between SKIP-RUN (wild-type or Δ177–186) and Arl8b or Rab1A as revealed by yeast two-hybrid assays. Yeast cells containing pGBD-C1-Arl8b(Q75L)ΔΝ or pGBD-C1-Rab1A(Q70L)ΔCys and pAct2-SKIP-RUN (wild-type or Δ177–186) were streaked on SC-AHLW and incubated at 30°C for 1 week. (B) Interaction between SKIP-RUN (wild-type or Δ177–186) and Arl8b or Rab1A as revealed by coimmunoprecipitation assays. Beads coupled with either FLAG-Arl8b(Q75L) (lanes 1–4) or FLAG-Rab1A(Q70L) (lanes 5–8) were incubated with COS-7 cell lysates containing T7-SKIP-RUN (lanes 1, 2, 5, and 6) or T7-SKIP(Δ177–186)-RUN (lanes 3, 4, 7, and 8), and the proteins bound to the beads were analyzed by immunoblotting with the antibodies indicated. (C) Typical images of melan-a cells expressing EGFP-SKIP(Δ177–186) together with Arl8b(Q75L)-mStr (top row), or mStr-Rab1A(Q70L) (bottom row) are shown (the lysosomal marker Lamp-1 images and their corresponding bright-field images). Cells exhibiting perinuclear melanosome aggregation are outlined with a broken line. The insets show magnified views of the boxed areas. The arrowheads point to the site of lysosome accumulation at the cell periphery. Note that melan-a cells expressing EGFP-SKIP(Δ177–186) together with mStr-Rab1A(Q70L) no longer exhibited peripheral melanosome accumulation (bottom row), whereas cells expressing Arl8b(Q75L)-mStr (top row) still exhibited peripheral lysosome accumulation. Scale bars, 20 μm. (D) The results are expressed as the percentages of transfected cells that exhibited peripheral melanosome accumulation, and the bars represent the means and S.E. of the data from three independent experiments (n >50). **, *p* <0.01 in comparison with the cells expressing both mStr-Rab1A(Q70L) and EGFP-SKIP (Student's unpaired *t* test).

To directly verify our hypothesis that Rab1A is a functional cargo receptor for SKIP–kinesin-1 in anterograde melanosome transport, we attempted to generate a SKIP mutant that specifically lacked Rab1A binding activity but retained Arl8 binding ability. To do so, we prepared a series of C-terminal truncation mutants of the SKIP-RUN and evaluated their binding abilities toward Arl8b and Rab1A by yeast two-hybrid assays (Supplementary Fig. S2A). Although the Arl8b-binding region and Rab1A-binding region of SKIP almost completely overlapped, close inspection of the results of the yeast two-hybrid assays indicated that amino acid residues 177–186 of SKIP appeared to be required for Rab1A binding, but not for Arl8b binding (Supplementary Fig. S2A). Fortunately, as revealed by both yeast two-hybrid assays and coimmunoprecipitation assays in COS-7 cells, deleting this small region (named Δ177–186) created an ideal mutant that specifically lacked Rab1A binding ability but retained Arl8b binding activity (Fig. 6A, B). When we coexpressed the SKIP(Δ177–186) mutant with constitutive active Arl8b(Q75L) in melanocytes, they colocalized with the lysosomes and still induced peripheral lysosome accumulation (Fig. 6C, top row), indicating that the SKIP(Δ177–186) mutant can recruit kinesin-1 to lysosomes in melanocytes. Coexpression of SKIP(Δ177–186) with Rab1A(Q70L), on the other hand, no longer induced peripheral melanosome accumulation (Fig. 6C, bottom row, and D), and the melanosomes aggregated around the nucleus instead. A similar melanosome aggregation phenotype also resulted when Rab1A(Q70L) alone^13^ was expressed, but the exact molecular basis for the phenotype remains unclear. Taken together, these results strongly indicated that the interaction between Rab1A and SKIP is essential for anterograde melanosome transport.

## Discussion

Melanosomes are often used as a model for analyzing organelle transport because of their visibility and highly dynamic movements along the two components of the cytoskeleton^35^. In contrast to the molecular machinery of retrograde melanosome transport and actin-dependent melanosome transport, the molecular mechanism of anterograde melanosome transport, especially the transport motor complex, had never been elucidated in mammalian melanocytes. In the present study, we discovered involvement of a transport complex composed of Rab1A, SKIP, and kinesin-1 in anterograde melanosome transport in melanocytes, in which Rab1A first recruits the Rab1A effector SKIP to the melanosome via binding to the N-terminal RUN domain-containing region of SKIP (Figs. 1A, 4, and 5B, C). Rab1A then indirectly recruits a kinesin-1 complex composed of Kif5b and KLC2 to the same melanosome through interaction between SKIP and KLC2. The resulting Rab1A–SKIP–kinesin-1 complex mediates anterograde melanosome transport in melanocytes, and thus loss of any one of the components of the complex results in the same perinuclear melanosome aggregation phenotype (Figs. 1–3)^13^. Conversely, coexpression of both SKIP and Rab1A(Q70L) in melanocytes often resulted in a novel melanosome distribution phenotype, named “peripheral melanosome accumulation”, in which melanosomes predominantly accumulated at cell protrusions (Fig. 5B, D), most likely by facilitating anterograde transport.

Another important finding in this study was the existence of two types of SKIP–kinesin-1 complexes in melanocytes: the Arl8b–SKIP–kinesin-1 complex on lysosomes (Supplementary Fig. S3) and the Rab1A–SKIP–kinesin-1 on melanosomes (Fig. 5). Because both Rab1A and Arl8b bind to almost the same region of the SKIP N-terminal region (Supplementary Fig. S2A) and Rab1A and Arl8b compete with each other (Fig. 5A), Rab1A and Arl8b function as independent cargo receptors for the SKIP–kinesin-1 complexes that regulate anterograde melanosome transport and lysosome transport, respectively.

Although Arl8b has recently been reported to be localized at lytic granules, another type of LRO, in natural killer cells and to regulate their motility by forming a complex with SKIP and kinesin-1, the same as in lysosome transport^36^, unlike the melanosomes that coexist with conventional lysosomes in melanocytes, the lytic granules in natural killer cells are likely to be wholly transformed lysosomes that carry out both specialized and universal functions^1^. It is therefore tempting to speculate that natural killer cells do not require two cargo receptors, one for specific transport of lysosomes and the other for specific transport of lytic granules (i.e., transformed lysosomes). It would be interesting to determine whether the 2-cargo receptor mechanism identified in this study operates in other types of cells, in which LROs and bona fide lysosomes are both present, e.g., platelets, which contain α and dense granules as well as lysosomes, and endothelial cells, which contain both Weibel-Palade bodies and lysosomes^1^.

Although Rab1A was originally reported as a Golgi-resident Rab that regulates ER-to-Golgi transport^37^,^38^, recent studies have shown that Rab1A also regulates other types of intracellular trafficking events, e.g., the motility of early endocytic vesicles^39^,^40^,^41^,^42^. Because the proteins found to be involved in the Rab1A-dependent anterograde transport system in this study (i.e., SKIP, KLC2, and Kif5b) are thought to be broadly expressed in mammalian cells^14^,^18^,^43^, it seems highly possible that the Rab1A–SKIP–kinesin-1 complex that we identified in this study regulates the transport of other intracellular vesicles besides melanosomes. Further investigation will be necessary to identify the other functions of this motor complex in intracellular vesicle transport.

In conclusion, we have discovered the molecular mechanism of microtubule-dependent anterograde melanosome transport, which was the sole remaining unknown melanosome transport mechanism in mammalian melanocytes. Our findings regarding the two independent cargo receptor mechanisms involved in the transport of lysosomes and LRO melanosomes provide new insights into the mechanisms by which cells use the same motor protein to differentially regulate the transport of two related organelles.

## Methods

### Materials

The following antibodies used in this study were obtained commercially: anti-β-actin mouse monoclonal antibody (clone: G043) (ABM, Richmond, BC, Canada); anti-Arl8b rabbit polyclonal antibody (GeneTex, Irvine, CA); anti-GFP (green fluorescent protein) rabbit polyclonal antibody (MBL, Nagoya, Japan); anti-KHC (kinesin heavy chain) mouse monoclonal antibody (clone: H2) and HRP-conjugated anti-T7 tag mouse monoclonal antibody (Merck Millipore, Billerica, MA); anti-KLC2 goat polyclonal antibody (Santa Cruz Biotechnology, Santa Cruz, CA); anti-Lamp-1 rat monoclonal antibody (clone: 1D4B) (BD Biosciences, San Jose, CA); anti-SKIP rabbit polyclonal antibody (Abcam, Tokyo, Japan); anti-Rab1A rabbit polyclonal antibody (Abnova, Taipei, Taiwan); and HRP (horseradish peroxidase)-conjugated anti-FLAG tag mouse monoclonal antibody (clone: M2) (Sigma-Aldrich, St. Louis, MO).

### Plasmid construction

cDNA of human SKIP was prepared as described previously^25^ and subcloned into the pmStr-C1 vector^11^. pEGFP-C1-SKIP was prepared as described previously^25^. cDNA of an shRNA-resistant (SR) form of SKIP was constructed by conventional PCR techniques using the following oligonucleotides (substituted nucleotides are shown in italics) 5′-ATGGAGCCGGGGGAGGTGAAGGA*T*CGGAT*T*CT*A*GAGAA*T*ATCTCG-3′ and subcloned into the pEGFP-C1 vector (BD Biosciences Clontech, Mountain View, CA). cDNA of SKIP-RUN (amino acid residues 1–282) was prepared as described previously^25^ and subcloned into the pEF-T7^44^, pEF-T7-GST^7^ and pAct2 (BD Biosciences Clontech) vectors. cDNAs of SKIP(Δ177–186) deletion mutants (full-length and RUN) were prepared by inverse PCR^45^ using a SKIP-RUN-harboring vector as a template and the following pair of oligonucleotides: forward primer, 5′-CACGGCTCAGACAGTCTGTCC-3′ and reverse primer, 5′-CAGCAGGTACTGAGGTTTGTAG-3′, and subcloned into the pEGFP-C1, pEF-T7 and/or pAct2 vectors. pSilencer-CMV-mStr vector^46^ expressing mouse *SKIP* shRNA was prepared essentially as described previously^47^ (19-base target site: 5′-GGACCGAATCCTGGAGAAC-3′). Other SKIP primers that were used to construct of plasmids for yeast two-hybrid assays are summarized in Supplementary Fig. S2B. cDNA of mouse Arl8b was amplified by PCR using melan-a cDNA as a template and the following pairs of oligonucleotides (restriction enzyme sites are underlined, and a kozak sequence and a stop codon are shown in italics and boldface, respectively): forward primer, 5′-GGATCCG*CCACCATG*CTGGCGCTCATCTCC-3′ and reverse primer, 5′-GTCGACGATCTCCGGGATTTTGAGTGTTG-3′ or 5′-**TCA**GCTTCTCCGGGATTTTG-3′. A GTPase activity-deficient mutant of Arl8b (Q75L, Gln-to-Leu substitution at amino acid position 75) was produced by the two-step PCR technique^48^ using the following mutagenic oligonucleotides (substituted nucleotides are shown in italics): forward primer, 5′-GACATTGGGGGGC*T*GCCCCGTTTCCGA-3′ and reverse primer, 5′-TCGGAAACGGGGC*A*GCCCCCCAATGTC-3′. The resulting Arl8b cDNAs were subcloned into the pmStr-N1^49^ and/or pEF-FLAG^44^ vectors. cDNA of Arl8b(Q75L) lacking the first 17 amino acid residues was constructed by conventional PCR techniques using the following oligonucleotides (restriction enzyme sites are underlined, and a stop codon is shown in boldface): forward primer, 5′-GGATCCAAGGAGGAGATGGAACTGAC-3′ and reverse primer, 5′-**TCA**GCTTCTCCGGGATTTTG-3′, and then subcloned into the pGBD-C1 vector^23^. cDNA of mouse Rab1A(Q70L) was prepared as described previously^24^ and subcloned into the pEF-FLAG vector. pmStr-C1-Rab1A(Q70L) and pEGFP-C1-Rab1A(Q70L) were prepared as described previously^13^. The sequence information regarding the oligonucleotides used to construct the tail domains of the mouse Kifs is available from the authors on request. siRNAs against mouse *Kif5b* no. 1 (19-base target site: 5′-TGACCAGAATTCTTCAAGA-3′) and *Kif5b* no. 2 (19-base target site: 5′-GCAGTCAGGTCAAAGAACA-3′) were chemically synthesized by Nippon Gene Co., Ltd. (Toyama, Japan). siRNAs against mouse *KLC2* no. 1 (cat no. 1376723) and *KLC2* no. 2 (cat no. 1376725) were chemically synthesized by Bioneer Co., Ltd. (Daejeon, Korea).

### Immunofluorescence

The immortal mouse melanocyte cell line melan-a, derived from a black mouse (generous gift of Dorothy C. Bennett, St. George's Hospital Medical School, London, UK), was cultured on glass-bottom dishes (35 mm dish; MatTek, Ashland, MA) as described previously^50^,^51^. Plasmids were transfected into melan-a cells by using FuGENE 6 (Promega, Madison, WI) or Lipofectamine 2000 reagents (Invitrogen Corp., Carlsbad, CA), each according to its manufacturer's instructions. Two or three days after transfection, the cells were fixed in 4% paraformaldehyde, permeabilized with 0.05% saponin (Sigma-Aldrich) and 0.5% BSA in PBS, and stained with anti-Lamp-1 antibody (1/1000 dilution) and/or anti-GFP antibody (1/1000 dilution). The antibodies were visualized with anti-rat Alexa Fluor 488/633-conjugated IgG and/or anti-rabbit Alexa Fluor 488-conjugated IgG (Invitrogen Corp.), and the cells were examined for fluorescence with a confocal fluorescence microscope (FV500; Olympus, Tokyo, Japan). The images were processed with Adobe Photoshop software (CS4).

### Melanosome distribution assays

Melanosome distribution was assessed by examining images of transfected melan-a cells that had been obtained at random (more than 50 cells/dish from three independent dishes). The perinuclear melanosome aggregation phenotype was identified as described previously^51^. Peripheral melanosome accumulation was recorded when more than 70% of the melanosomes were present at the periphery of the cell. The data are expressed as means and standard error (S.E.). Student's unpaired *t* test or Dunnett's test (for multiple comparisons) was used to perform the statistical analyses, and *p* values <0.05 were considered statistically significant.

### Immunoblotting

Melan-a cells that had been seeded in 60 mm culture dishes the day before transfection were transfected with pSilencer vectors by using Lipofectamine 2000 according to the manufacturer's instructions. Two days after transfection, the cells were passaged to another 60 mm culture dish and then transfected with the same pSilencer vectors again. This procedure was repeated once again (a total of three transfections). Eight days after the first transfection, the cells were lysed with a lysis buffer (50 mM HEPES-KOH, pH7.2, 150 mM NaCl, 1 mM MgCl_2_, and 1% Triton X-100 supplemented with complete EDTA-free protease inhibitor cocktail (Roche Applied Science)), and the lysates were analyzed by 5% and 10% SDS-PAGE followed by immunoblotting with specific antibodies. siRNAs were transfected into melan-a cells, and at 72 hours after transfection the cells were harvested and lysed with the lysis buffer. Total cell lysates were analyzed by 7.5% SDS-PAGE followed by immunoblotting with specific antibodies.

### *In vitro* binding assays

Coimmunoprecipitation assays in COS-7 cells were performed with agarose beads conjugated with anti-FLAG tag antibody (Sigma-Aldrich) as described previously^44^,^52^. Immunoprecipitated proteins and coimmunoprecipitated proteins were analyzed by 10% SDS-PAGE followed by immunoblotting with specific antibodies.

Competition experiments between Rab1A and Arl8b were performed with glutathione Sepharose 4B beads coupled with T7-GST-SKIP-RUN (GE Healthcare Ltd., Little Chalfont, UK) essentially as described previously^53^. In brief, T7-GST-SKIP-RUN was transiently expressed in COS-7 cells and affinity-purified with glutathione Sepharose beads. Beads coupled with T7-GST-SKIP-RUN or beads alone were incubated for 3 hours at 4°C with COS-7 cell lysates containing FLAG-Rab1A(Q70L) and/or FLAG-Arl8b(Q75L). The proteins bound to the beads were analyzed by 11.25% SDS-PAGE followed by immunoblotting with specific antibodies. The intensity of the immunoreactive bands in Fig. 5A was measured with ImageJ software.

### Yeast two-hybrid assays

Yeast two-hybrid assays were performed by using pGBD-C1 vectors that harbor constitutively active/constitutively negative (CA/CN) Rab mutants lacking the C-terminal geranylgeranylation site (ΔCys) or Arl8b(Q75L) lacking the N-terminal acetylation site (ΔN; deletion of the first 17 amino acid residues) and pAct2 vectors that harbor the tail domains of all of the Kifs (i.e. tail domains of Kif1–27 and Kifc1–3) or SKIP RUN domain (see also Supplementary Figs. S1, S2) as described previously^22^,^24^,^54^. The yeast strain (pJ69-4A), selection medium SC-AHLW (synthetic complete medium lacking adenine, histidine, leucine, and tryptophan), culture conditions, and transformation protocol used were as described elsewhere^23^.

### Coimmunoprecipitation assays in melan-a cells

Protein-A Sepharose beads were incubated overnight at 4°C with either the anti-Rab1A rabbit polyclonal antibody described above or control rabbit IgG, and then with melan-a cell lysates overnight at 4°C. After washing the beads three times with 1 ml of the washing buffer, the proteins bound to the beads were analyzed by SDS-PAGE (5%, 7.5%, or 10% gel) followed by immunoblotting with the antibodies indicated in Fig. 4.

### Live cell imaging

Melan-a cells that had been cotransfected with both pEGFP-C1-SKIP and pmStr-C1-Rab1A(Q70L) were maintained at 37°C under 10% CO_2_ in an incubator (Olympus), and images of living cells were acquired at 1.0-second intervals with a time-lapse microscope (FV1000-D, Olympus).

### RT (reverse transcription)-PCR analysis

The total RNA of melan-a cells was prepared with TRI-reagent (Sigma-Aldrich), and reverse transcription was performed by using ReverTra Ace® (Toyobo, Osaka, Japan) according to the manufacturer's instructions. The pairs of oligonucleotides used for amplification were: for *Kif5a*, forward primer, 5′-ATCTGCGTTGTGAGCTTCCT-3′ and reverse primer, 5′-CTGGTCCTCAGCCTCATAGC-3′; for *Kif5b*, forward primer, 5′-GGATCCGATAAACCAGCTGCTGCAGT-3′ and reverse primer, 5′-TCAAGTTCCTTCTGGTTGCTTCA-3′; for *Kif5c*, forward primer, 5′-CCTGAACCTGCTTCTCAAGG-3′ and reverse primer, 5′-CTCGTCAGGTGCTCCTTTTC-3′; and for *GAPDH* (glyceraldehyde-3-phosphate dehydrogenase), forward primer, 5′-ACCACAGTCCATGCCATCAC-3′ and reverse primer, 5′-TCCACCACCCTGTTGCTGTA-3′; black/white inverted images of ethidium bromide-stained gels are shown.

## Author Contributions

M.I., N.O. and M.F. conceived and designed the study; M.I. and M.F. performed the experiments; M.I., N.O. and M.F. prepared the manuscript.

## Supplementary Material

Supplementary Information Supplementary figures S1-S3
