# Supplementary material for: Rab1A regulates anterograde melanosome transport by recruiting kinesin-1 to melanosomes through interaction with SKIP
Source: Sci Rep. 2015 Feb 4;5:8238. doi: 10.1038/srep08238 (PMC4316160; doi:10.1038/srep08238)
Supplement: Supplementary Information — Supplementary figures S1-S3 [file srep08238-s1.pdf]

Supplementary Figures

Rab1A regulates anterograde melanosome transport by recruiting kinesin-1 to melanosomes through interaction with SKIP

Morié Ishida, Norihiko Ohbayashi & Mitsunori Fukuda

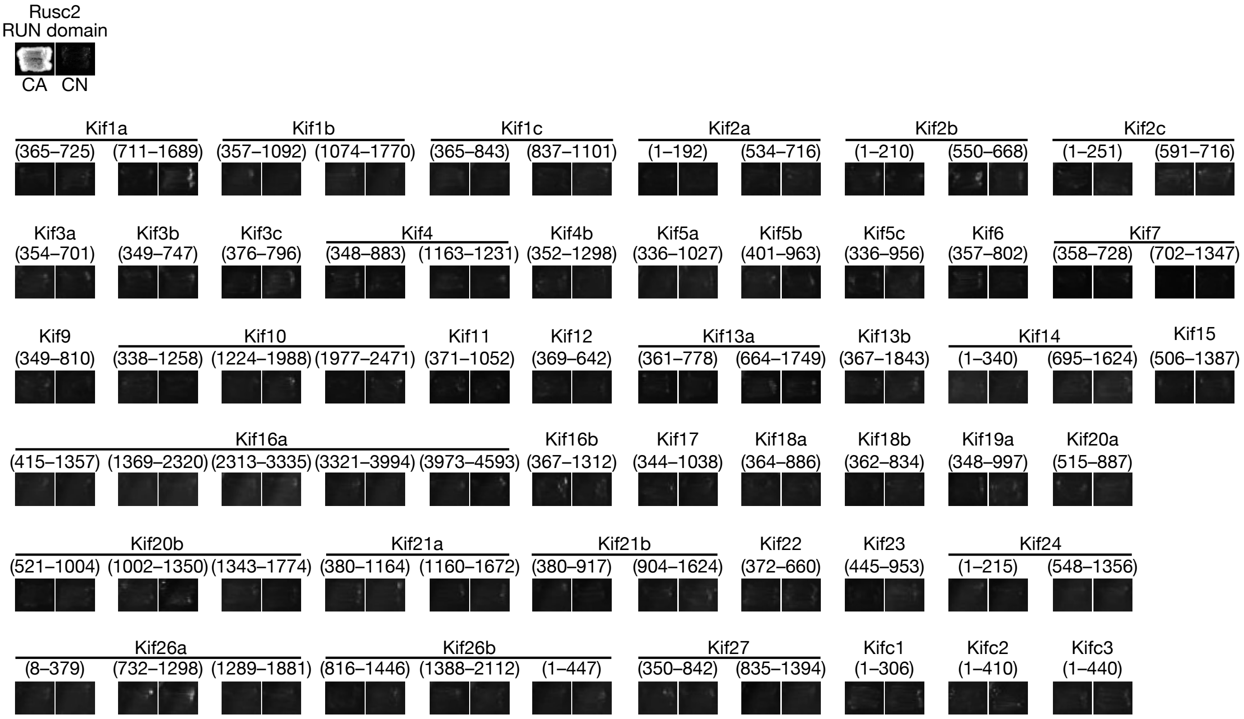

Supplementary Figure S1. Yeast two-hybrid screening for tail domains of Kifs that interact with Rab1A. Yeast cells containing pGBD-C1-Rab1A constitutively active/constitutively negative (CA/CN) mutants (left/right panel) and pAct2 plasmids expressing tail domains of mouse Kifs (i.e., Kif1–27, Kifc1–3) were streaked on SC-AHLW and incubated at 30°C for 1 week. Interaction between the Rusc2 RUN domain and Rab1A(CA) is shown as a positive control[25]. Note that none of the tail domains of the Kifs interacted with Rab1A(CA/CN).

A

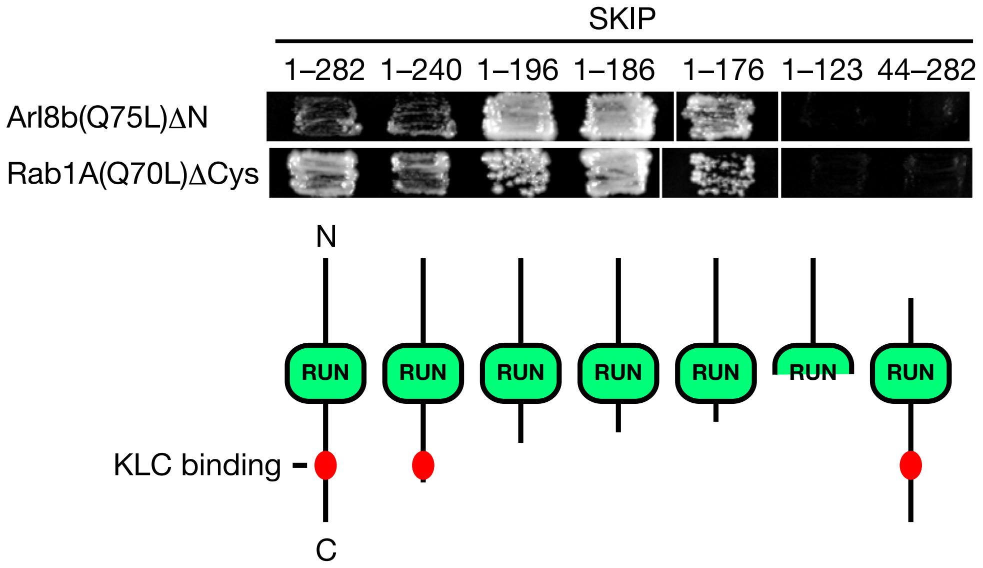

B

pAct2-SKIP	Forward primers	Reverse primers
1–282	5’-AGATCTATGGAGCCGGGGGAGGTGAA-3’	5’-**TCA**GGAGGACACAGTCTCTGCCG-3’
1–240		5’-**TCA**GTCTCCATCTTCCCAGTCTG-3’
1–196		5’-**TCA**AGAGTTGAGGGACAGAC-3’
1–186		5’-**CTA**GACCGAGCTGGGAAGGCG-3’
1-176		5’-**CTA**CAGCAGGTACTGAGGTTTG-3’
1–123		5’-**CTA**GTACTTATGCAGCAGGC-3’
44–282	5’-AGATCTCGTCTGTGTGAGCACCTGGA-3’	5’-**TCA**GGAGGACACAGTCTCTGCCG-3’

Supplementary Figure S2. Rab1A binding activity and Arl8b binding activity of the SKIP N-terminal deletion constructs. (A) Interaction between SKIP deletion mutants and Arl8b or Rab1A as revealed by yeast two-hybrid assays. Schematic representations of the SKIP constructs used in these assays are shown at the bottom (RUN domain and kinesin light chain (KLC) binding region[18] are shown in green and red, respectively). (B) List of the primers used to generate the SKIP mutants in (A) (restriction enzyme sites are underlined and the stop codon is shown in boldface).

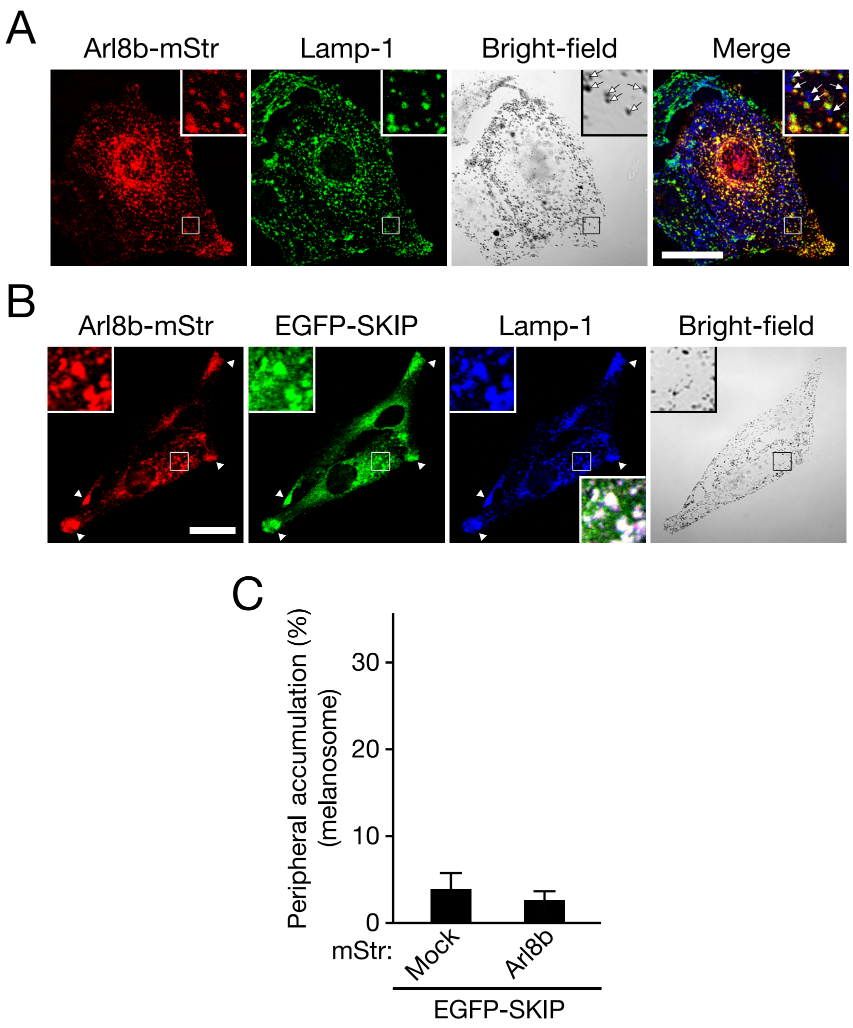

Supplementary Figure S3. Arl8b is localized at the lysosomes in melanocytes and regulates anterograde lysosome transport through interaction with SKIP. (A) Typical images showing localization of Arl8b-mStr in melan-a cells stained for the lysosomal marker Lamp-1. The melanosomes in the merged images (far right panel) are pseudocolored blue. Note that Arl8b is clearly localized at the lysosomes and not localized at the melanosomes (far right panel). The arrows point to melanosomes that are negative for both Arl8b and Lamp-1. (B) Typical images of melan-a cells expressing EGFP-SKIP together with Arl8b-mStr are shown (the lysosomal marker Lamp-1 images and their corresponding bright-field images). The insets are magnified views of the boxed areas. The lower inset in the second panel from the right is a merged image of Arl8b-mStr, EGFP-SKIP, and Lamp-1. The arrowheads point to the site of lysosome accumulation at the cell periphery. Note that cells expressing EGFP-SKIP together with Arl8b-mStr exhibited peripheral lysosome accumulation and a normal melanosome distribution. Scale bars, 20 μm. (C) Quantification of the results shown in (B). The results are expressed as the percentages of transfected cells that exhibited peripheral melanosome accumulation, and the bars represent the means and S.E. of the data from three independent experiments (n >50).
